# 3D printing of nanocomposite pills through desktop vat photopolymerization (stereolithography) for drug delivery reasons

**DOI:** 10.1186/s41205-022-00130-2

**Published:** 2022-01-17

**Authors:** Peeyush Kumar Sharma, Dinesh Choudhury, Vivek Yadav, U. S. N. Murty, Subham Banerjee

**Affiliations:** 1 Department of Pharmaceutics, National Institute of Pharmaceutical Education & Research (NIPER)-Guwahati, Changsari, Assam 781101 India; 2 National Centre for Pharmacoengineering, NIPER-Guwahati, Changsari, Assam 781101 India; 3 NIPER-Guwahati, Changsari, Assam 781101 India

**Keywords:** Vat photopolymerization, 3D printing, Nanocomposites, Drug delivery

## Abstract

**Background:**

The desktop vat polymerization process or stereolithography printing is an ideal approach to develop multifunctional nanocomposites wherein a conventional solid dosage form is used as a reservoir for compliant administration of drug-loaded nanocarriers.

**Methods:**

In this study, a nanocomposite drug delivery system, that is, hydrogel nanoparticles of an approved nutraceutical, berberine entrapped within vat photopolymerized monoliths, was developed for drug delivery applications. For the fabrication of the nanocomposite drug delivery systems/pills, a biocompatible vat photopolymerized resin was selected as an optimum matrix capable of efficiently delivering berberine from stereolithography mediated 3D printed nanocomposite pill.

**Results:**

The obtained data reflected the efficient formation of berberine-loaded hydrogel nanoparticles with a mean particle diameter of 95.05 ± 4.50 nm but low loading. Stereolithography-assisted fabrication of monoliths was achieved with high fidelity (in agreement with computer-aided design), and photo-crosslinking was ascertained through Fourier-transform infrared spectroscopy. The hydrogel nanoparticles were entrapped within the pills during the stereolithography process, as evidenced by electron microscopy. The nanocomposite pills showed a higher swelling in an acidic environment and consequently faster berberine release of 50.39 ± 3.44% after 4 h. The overall results suggested maximal release within the gastrointestinal transit duration and excretion of the exhausted pills.

**Conclusions:**

We intended to demonstrate the feasibility of making 3D printed nanocomposite pills achieved through the desktop vat polymerization process for drug delivery applications.

## Background

Additive manufacturing or three-dimensional printing (3DP) is one of the most advanced manufacturing technique for producing customized products [1, 2]. Recently, 3DP technology has emerged as an extremely promising approach for achieving 3D printed objects with excellent resolution and customizability [1]. The primary step of 3DP is designing a computer-aided design (CAD) model of the required object with a defined size and shape. The CAD model was transferred to a 3D printer software, where the model image was algorithmically sliced into individual layers, and the 3D model was formed by additive consolidation of the individual layers [2].

3DP has been successfully translated into a multitude of applications across the industrial spectrum, including drug formulation technology [3–5]. 3DP has gained significant traction in formulation technology for prototyping drug dosage forms with complex geometries and compositions [6, 7]. Pioneering work from Basit and Lamprou provided it a central stage in drug delivery and pharmaceutics. This technology has been explored in controlled oral delivery systems [8–11], transdermal and topical delivery [12], implant materials and medical devices [13], and multidrug and multifunctional drug delivery devices [4, 14–16].

Across the various categories of 3DP technology, such as fused deposition modelling (FDM) [17], selective laser sintering [18], and stereolithography (SLA), the latter stands out as a vat polymerization technique wherein a photo-crosslinkable resin liquid is converted into a solid upon light irradiation [19]. Precise spatial control over irradiation provides excellent resolution and accuracy [7, 19]. A wide array of drug delivery systems ranging from microneedles [20, 21] to polypills [22] and personalized devices has been reported in recent literature [4, 21, 23–25].

By virtue of this liquid-to-solid transformation, SLA can be an excellent prospect for the manufacture of nanocomposite systems [26, 27]. A variety of examples are available in the literature wherein SLA 3D printed nanocomposites were prepared to improve the material properties of the 3D printed constructs [28–30]. The ability of this conjugation opens avenues for controlled drug delivery applications of such constructs. In the current study, we used SLA to produce a drug containing nanocomposite pills through vat photopolymerization-mediated desktop 3D printing. To the best of our knowledge, a nanocomposite pill constructed using the vat photopolymerization (SLA) technique has not yet been reported for drug delivery.

Berberine (BBR) is a natural alkaloid found in several plants [31]. BBR has a wide range of pharmacological activities, including antimicrobial, antiprotozoal, antidiabetic, and anticancer activities [32]. Due to its antiprotozoal activity, it has been proven to be efficacious against leishmaniasis. Different in vitro studies have revealed the potential of BBR in treating leishmaniasis by inhibiting multiplication, respiration, and anabolism of defined amastigote phases [33]. BBR regulates lipid and glucose metabolism, oxidative stress, and inflammatory responses, making it a promising therapeutic drug for treating metabolic disorders, such as non-alcoholic fatty liver disease and diabetes [31]. BBR was selected in the present study as pharmacokinetic studies in rodents and humans have reported low gut absorption and rapid metabolism of BBR due to its self-aggregation under physiological conditions, thus requiring high doses of BBR to achieve optimal therapeutic efficacy. Therefore, efforts are being made to increase its gut absorption by enhancing its permeation or using P glycoprotein inhibitors (to inhibit the efflux of BBR) and by using lipid nanoparticle delivery systems [34]. Keeping in mind the delivery challenges, we envisaged a nanocarrier platform capable of facilitating the aqueous solubility and subsequent absorption of BBR. A nanocarrier with a charge-neutral polyethylene glycol (PEG) corona has been shown to improve the intestinal absorption of BBR owing to its mucopenetrating properties [35, 36]. The incorporation of PEG improves the solubility of BBR in aqueous environments, leading to better absorption and permeation [37]. However, the near-neutral surface charge of PEG can accelerate the settling down of the hydrogel nanoparticles, thus hampering the absorption process; therefore, we hypothesised that the immobilisation of BBR into the 3D printed pill system could provide a sustained release of hydrogel nanoparticles and better absorption over time. The nanocomposite approach can provide better patient adherence due to the oral unit dosage form and potentially improve the absorption and degradation challenges associated with BBR.

As the photo-crosslinking process in SLA is based on the free radical chain reaction, acrylate or methacrylate-based chemicals can be used [38, 39]. Hence, PEGDA was selected and it is widely used for making 3D prototypes in SLA based printer intended for drug delivery. In vitro study showed the biocompatible property of PEGDA to human cells [40]. PEO was used in resin to maintain the required viscosity of the resin and also as a swelling aid for the printed prototypes.

In the present work, an attempt was made to utilize vat polymerization (SLA) to develop BBR-loaded hydrogel nanoparticles (BBR-NPs) immobilized within a unit dose of nanocomposite monoliths. In this regard, a BBR-NP-loaded biodegradable resin composed of poly (ethylene glycol) diacrylate (PEGDA) as a photo-cross-linkable monomer and poly (ethylene oxide) (PEO) as a swelling aid, and SLA was utilized to obtain a nanocomposite drug delivery system. The pharmaceutical performances of the prepared units were evaluated.

## Methods

### Materials

Berberine chloride (BBR), poly (ethylene glycol) diacrylate (PEGDA; mol wt. 700 Da), poly (ethylene oxide) (PEO; mol wt. 100,000 Da), diphenyl (2,4,6-trimethylbenzoyl) phosphine oxide (TPO), and sodium persulfate (SPS) were purchased from Sigma-Aldrich Chemical Co. St. Louis, MO, USA. All other reagents used were of analytical grade and obtained from HiMedia Laboratories Pvt. Ltd. Solvents used in HPLC were of chromatography grade and obtained from Merck Life Sciences Pvt. Ltd. Double-distilled water (DDW) was used throughout the study.

### Preparation and characterizations of BBR-NPs

BBR-NPs were prepared using a combination of PEGDA, PEO, and the photoinitiator (TPO) with the process of photopolymerization [41] and characterised further for particle size and surface charge. The mean particle size, zeta potential, and polydispersity index of the BBR-NPs were evaluated using dynamic light scattering (Zeta Sizer Nano ZS, Malvern Instruments Ltd., UK) after diluting the samples with DDW (100x dilution). To determine the BBR content and entrapment efficiency, BBR-NPs were subjected to ultrafiltration through a 3 kDa ultrafiltration system, and the filtrate was dissolved in an organic mixture of methanol: acetonitrile (1:1) (10 mL) and stirred overnight to allow complete extraction of BBR from the NPs. An aliquot from the sample was filtered through a 0.22 μm syringe filter, diluted with ultrapure water, and analyzed using RP-HPLC as per a previously described method with slight modifications [42]. The chromatographic system consisted of auto-sampler mediated (WPS 3000 TSL ANALYTICAL) reverse-phase high-performance liquid chromatography (RP-HPLC, Ultimate 3000, Thermo Fisher Scientific, US) equipped with a quaternary pump (LPG-3400 RS, Smart flow™) and PDA detection system (DAD 3000) was used. This system was operated using Chromeleon® software (version 7.2.8) to control the instrument parameters. The column temperature was controlled in an oven (TCC 3000 SD) to accommodate the columns inside the chamber. A C-18 column (HYPERSIL GOLD™, 5 μm particle size ODS, 150 mm × 4.6 mm) was used throughout the elution process. The mobile phase was composed of an isocratic system of Acetonitrile/ 10 mM ammonium acetate containing 0.2% TEA at pH 5.0, adjusted using acetic acid at a ratio of 35:65. The flow rate was fixed at 1.00 mL/min with an injection volume of 5 μL. Drug content was calculated as the total drug per ml of the NP suspension. The encapsulation efficiency was calculated based on the difference in drug content between the separated NPs and the ultrafiltration supernatant.

### Fabrication of 3D printed nanocomposite pills

**Fig. 1:**
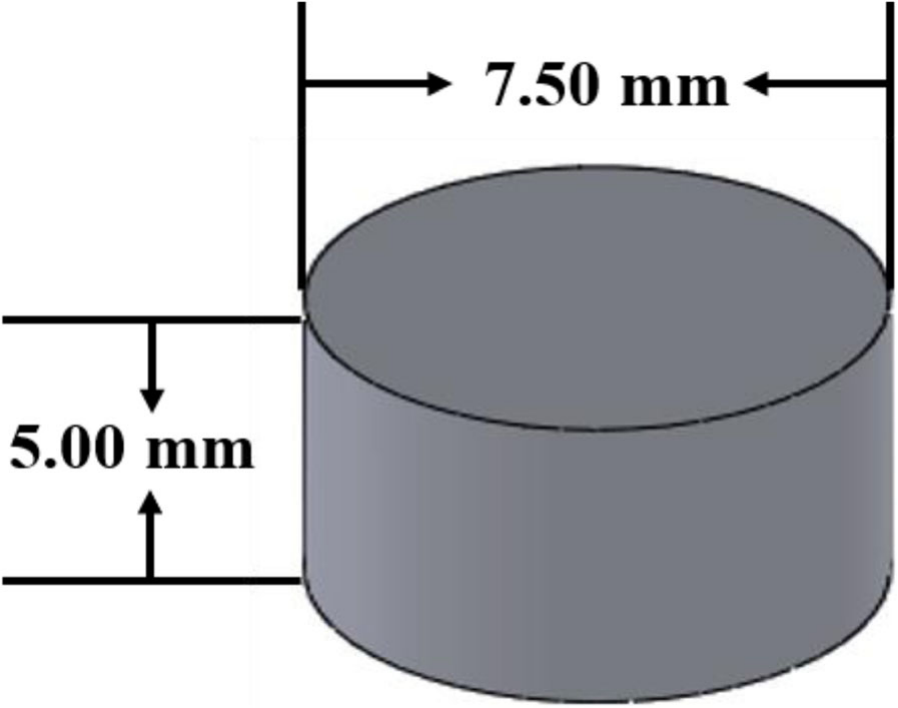
CAD designed 3D model of a nanocomposite drug delivery system with dimensions

The prepared BBR-NPs (after filtration using 0.45 μm filter) were suspended in the above-mentioned resin solution, maintaining the final composition of polymers and TPO as a photo initiator (PI) in the resin solution (25% PEGDA, 3% PEO 100 k, 0.03% TPO, 0.12% SPS). Here, PEO also acts as a viscosity enhancer and a suspending agent to ensure homogenous dispersion of BBR-NPs across the resin solution. The fortified resin solution was poured onto the resin tank of the Form 2 SLA printer (Formlabs, UK), and the templates used to print the nanocomposite units were designed using SolidWorks 2019 (Dassault Systems) and exported as a .stl file into the 3D printer software (Preform Software v. 1.9.1, Formlabs, UK). The printer was operated in open mode with a clear resin selected using Preform software. A 3D printed pill with 7.50 mm diameter and 5.00 mm thickness with a layer height of 50 μm was uploaded into the 3D printer (Fig. 1). Seven nanocomposite units were printed to achieve batch uniformity. The obtained nanocomposite units were rinsed in DI water to remove the unreacted resin from the surface.

### Physical characterization

Physical dimensions and the mean weight of the prepared nanocomposite units (*n* = 7) were assessed using a digital vernier caliper (CD-6″ ASX, Mitutoyo Corporation, Japan) and digital balance, respectively. Air-dried 3D printed nanocomposite drug delivery systems were also subjected to conventional friability and hardness testing to ascertain the mechanical resilience and commercial applicability of the nanocomposite drug delivery system. Seven air-dried nanocomposite drug delivery systems were selected for each test and introduced to the respective friability testing apparatus and hardness testing machine. The % weight loss was reported in friability studies, while transverse hardness was reported in hardness testing.

### Fourier transform infrared (FTIR) spectroscopy

The FTIR spectra of air-dried samples of the nanocomposite drug delivery system and BBR-NPs were obtained using an ATR spectrometer (ALPHA II, Bruker, Germany) along with the spectra of pristine resin components and BBR. Spectral changes upon polymerization and possible interactions are observed.

### Swelling studies

For gravimetric swelling studies, three nanocomposite units (air-dried to constant weight) were individually weighed, immersed in buffers of pH 1.2, and pH 6.8, and kept in a laboratory shaker at 37 °C. Each pill was removed from the buffer solutions at specific time points and weighed after removing the excess surface liquid. The process was repeated for up to seven days, and the gravimetric swelling of the nanocomposite units was reported. The amount of media absorbed by the hydrogels was measured gravimetrically and expressed in terms of swelling percentage using the equation given below [43]:

1 $$ \mathrm{Swelling}\ \mathrm{ratio}\ \left(\%\right)=\frac{Ws-{W}_0}{W_0}\times 100 $$

W_s_ is the weight of the swollen hydrogel at time t, and W_0_ is the initial weight.

### BBR content and loading

The drug content of the 3D printed nanocomposite pill was determined using an extraction method. The pill was crushed and placed in a flask filled with acetonitrile (10 mL) and allowed to stir for 24 h to ensure complete extraction of the drug from the pill. The aliquot was taken, diluted with ultrapure water, filtered through a 0.22 µm  syringe filter, and estimated using the above mentioned HPLC method.

### Scanning electron microscopy (SEM)

Air-dried samples to constant weight (samples were allowed to air dry for 48 h) were used for SEM analysis (FESEM, JEOL JSM-7610F, UK), and the surface and cross-sectional morphologies of the samples were prepared. The experimental samples were cut into small pieces and deposited onto a tape (NEM Tape, Nisshin Em. Co. Ltd. Tokyo, Japan), and a single-coated platinum coating was used. The experimental samples were then kept on a stub, and scanning was performed. The SEM images were captured at the required magnification at room temperature.

### In vitro BBR release

In vitro BBR release from the nanocomposite pills were conducted in gastric and intestinal pH conditions. (*n* = 3). The dialysis bag method was used for the drug release. The nanocomposite units were first introduced into HCL buffer (pH 1.2) for the first 4 h of the release experiment, after which the pill was transferred to a release medium mimicking intestinal conditions (phosphate buffer pH 6.8) and incubated for 48 h in a laboratory shaker at 37 °C and 100 rpm. Aliquots (1 mL) were removed from the release media at specific time points and replenished with fresh media [44]. The percentage of drug release was estimated using RP-HPLC. The percentage release data were then fitted to a variety of release kinetics to predict a plausible release kinetics model.

## Results

**Fig. 2:**
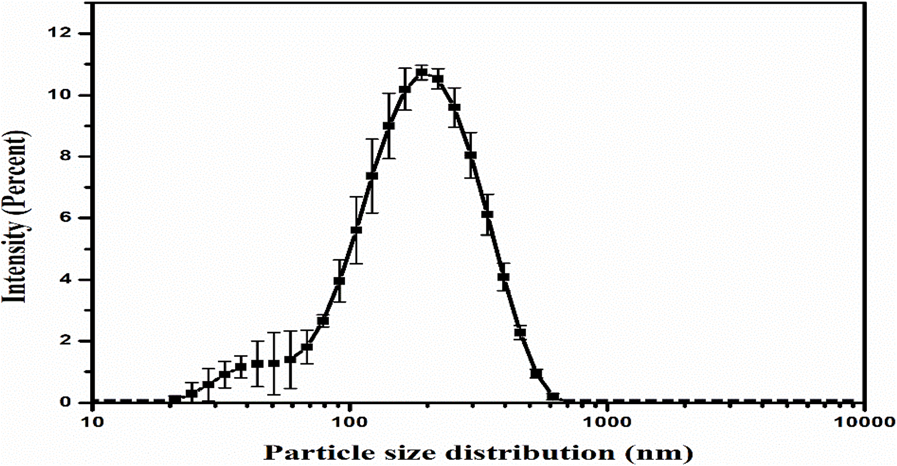
The particle size distribution of the prepared BBR-NPs by Dynamic light scattering (*n* = 3)

The nanoparticle suspension was subjected to ultrafiltration through a 0.45µm  filter under centrifugation at 3900 rpm to separate sub-0.45µm particles. The separated BBR-NPs were then subjected to particle size and zeta potential measurements. The BBR-NPs showed a mean particle diameter of 95.05 ± 4.50 nm with a polydispersity index of 0.312 ± 0.090 (Fig. 2.) and a mean zeta potential value of 0.378 ± 0.012 mV.

**Fig. 3:**
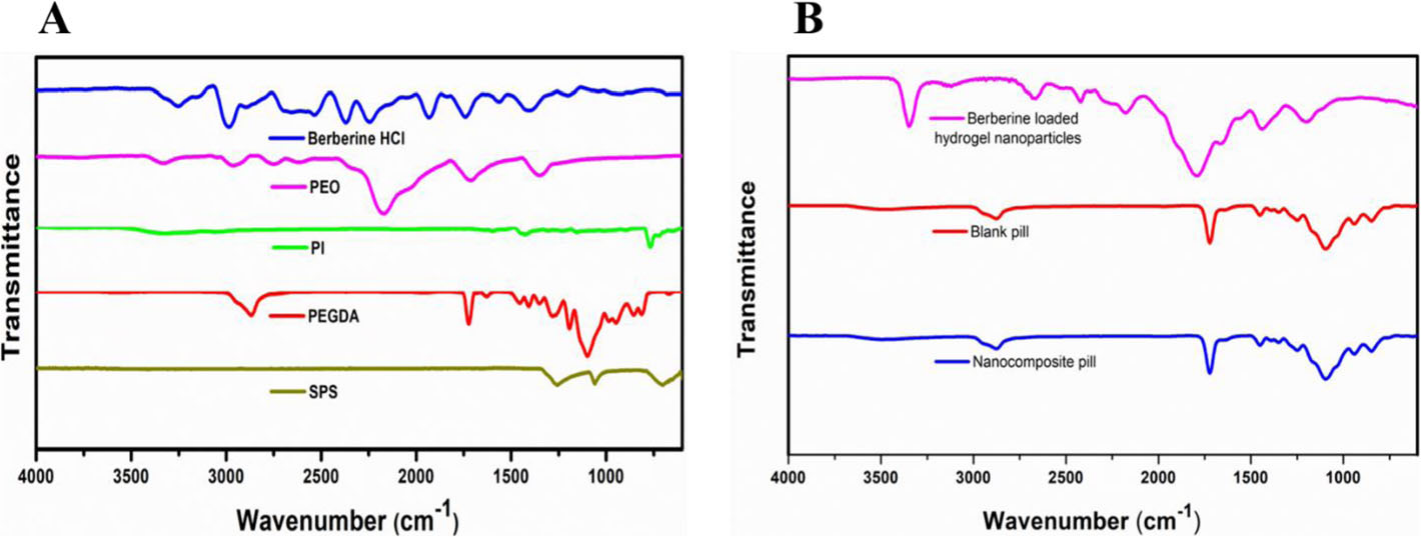
FTIR spectra of resin components and BBR (**A)** and 3D printed prototypes (**B)**

The BBR-NPs were subjected to ultrafiltration to separate the unloaded drug. For drug quantification studies; initially, the resin was prepared at a drug concentration of 1.5 mg/mL, and ultimately after the ultrafiltration [45] step for removing the free drug, the drug content was found to be 0.105 ± 0.09 mg/ml of BBR-NPs with an encapsulation efficiency of the desired population being 7.04 ± 0.34%. The BBR-NPs were air-dried and characterised for photocuring using FT-IR spectroscopy, with the disappearance of acrylate-specific peaks at 1402 cm^− 1^ and 1190 cm^− 1^ in the BBR-NPs, but can be seen in the FTIR spectra of pristine PEGDA. The disappearance of the acrylate peak occurred due to the crosslinking of PEGDA upon photopolymerization and simultaneous crosslinking. The strong peak at 1096 cm^− 1^ also confirms the presence of PEO in the BBR-NPs (Fig. 3).

**Fig. 4:**
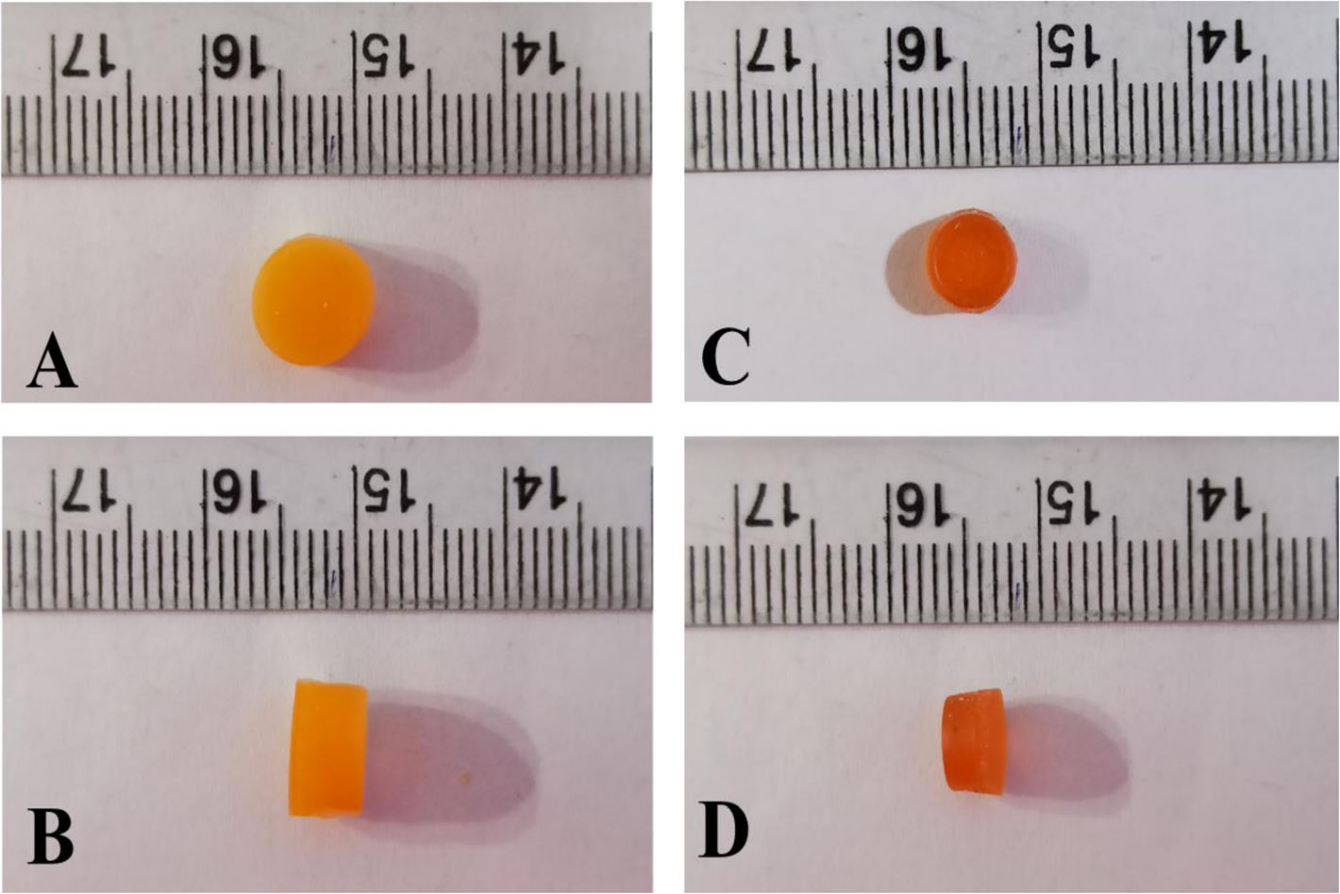
Dimensions of as printed and air-dried nanocomposite drug delivery system. Diameter and height of as printed (A-B) and air-dried (C-D) nanocomposite pill

After mixing the BBR-NPs suspension, the theoretical concentration of BBR in the resin mixture was 21.12 μg/ml. The resin solution was poured into the 3D printer, and nanocomposite units of the dimensions (diameter = 7.68 ± 0.15 mm and thickness = 5.01 ± 0.09) were printed with excellent printability and reproducibility (Table 1). The designed and printed dimensions of the nanocomposite drug delivery system, along with the mean dimensions and mean weights before and after air drying, are listed in Table 1. The printed nanocomposite drug delivery system exhibited substantial mass loss and volume loss (Table 1 and Fig. 4). The nanocomposite drug delivery system showed a percent friability of 0.063 ± 0.011, while the mean hardness of the nanocomposite units was found to be 37.90 ± 2.18 N.

**Table 1:**
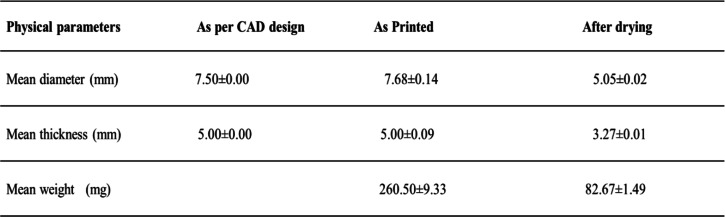
Dimensions and gravimetric specifications of as printed and air-dried nanocomposite drug delivery system

**Fig. 5:**
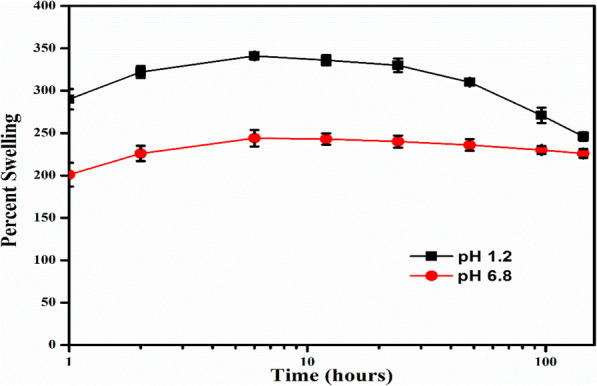
Swelling behaviour of the nanocomposite drug delivery system in gastric (pH 1.2) and intestinal (pH 6.8) environments

**Fig. 6:**
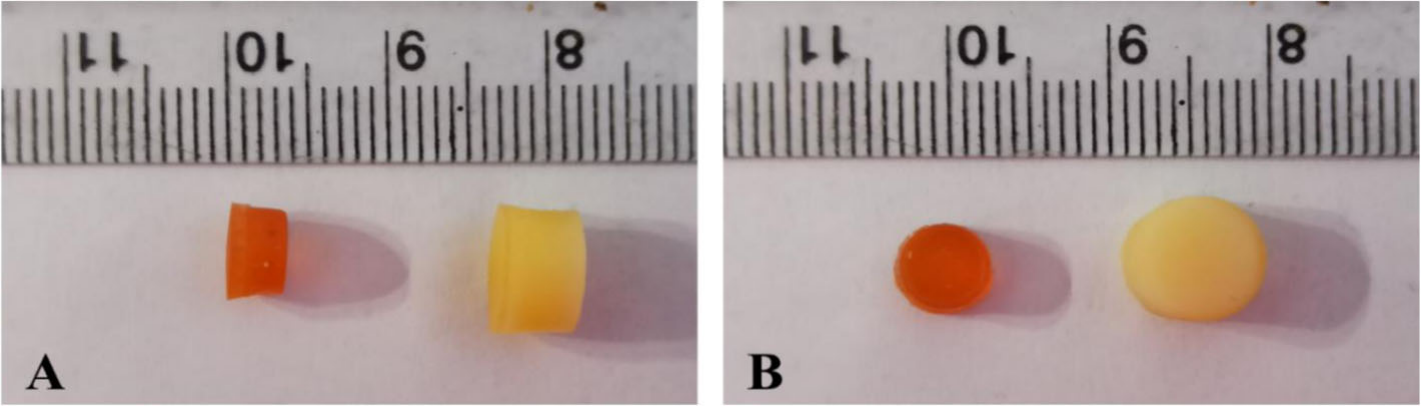
Volumetric swelling of the nanocomposite drug delivery system showing increase in height (A) and diameter (B) of the nanocomposite drug delivery system

The 3D printed nanocomposite drug delivery system was air-dried to a constant weight before conducting the swelling and release experiments. The maximum swelling was observed at pH 1.2, which was found to be 321.12 ± 12.22%, while at pH 6.8, the maximum swelling shown by the nanocomposite was 244.01 ± 9.51% (Fig. 5), and the digital images of the swollen nanocomposite are shown in Fig. 6.

Next, the prepared nanocomposite drug delivery system was subjected to drug content determination studies using the extraction method, with BBR HCl being introduced into the resin in the form of BBR-NPs. The drug content per nanocomposite pill was 3.670 ± 0.013 μg per unit. The drug content was lower than the theoretical value of 4.109 μg per pill (as calculated from the previously determined drug loading in hydrogel NPs).

**Fig. 7:**
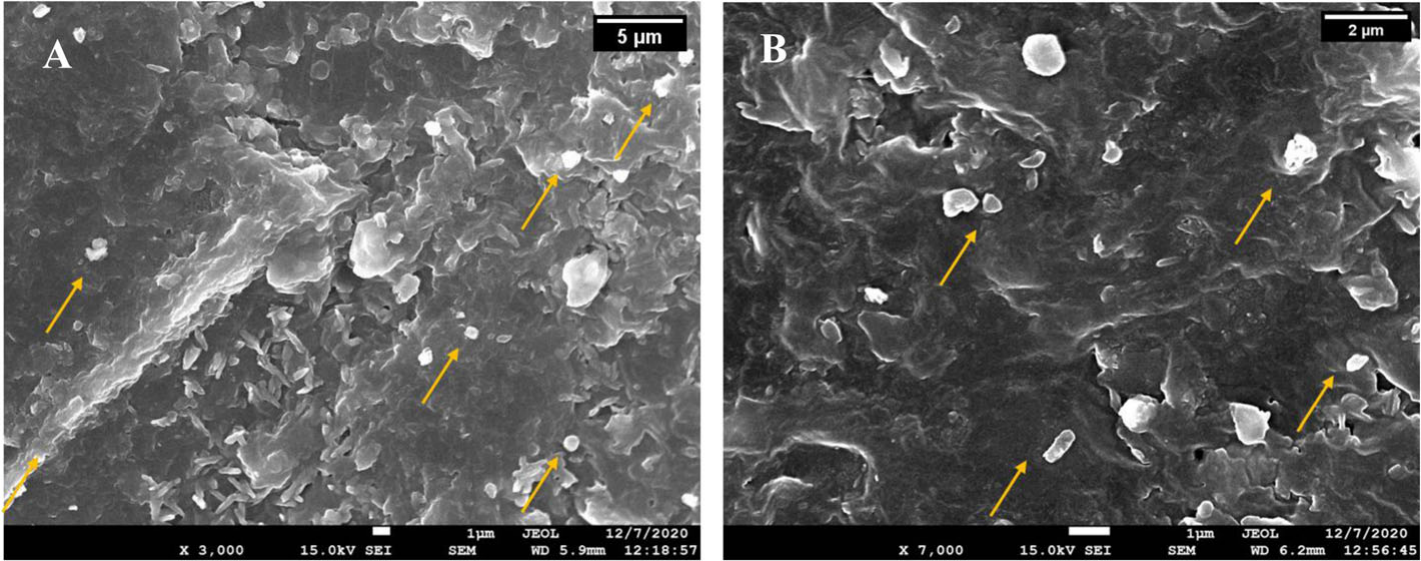
SEM images of the cross-sectional area of nanocomposite drug delivery system at two magnifications (A) 3000x and (B) 7000x (yellow arrows indicate the presence of BBR-NPs in the matrix)

The surface and cross-sectional morphologies of the prepared nanocomposite drug delivery system were observed using SEM, as shown in Fig. 7. The micrographs  confirmed the presence of BBR-NPs across the surface and cross-section surface of the nanocomposite drug delivery system (indicated by yellow arrows in Fig. 7), with sizes ranging from 100 nm to around a micron.

**Fig. 8:**
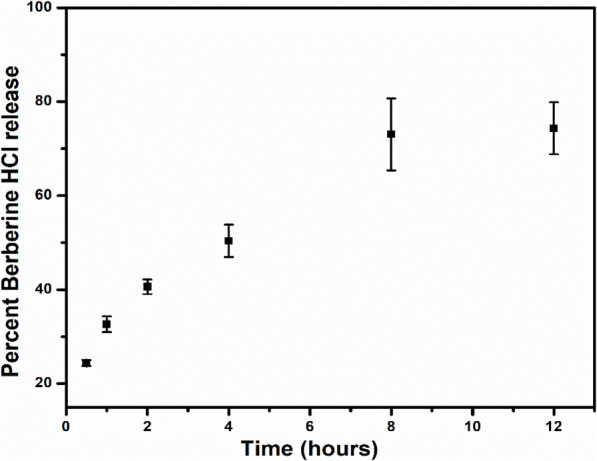
BBR HCl release profile from the 3D printed nanocomposite drug delivery system (*n* = 3). Initial 4 h in gastric media (pH 1.2) followed by intestinal media (pH 6.8) up to 48 h

The prepared nanocomposite drug delivery system was then subjected to in vitro drug release studies to understand the release behaviour in the gastrointestinal tract, and the cumulative release data were plotted against time. The release was initially observed in the stomach pH environment (pH 1.2) for an initial 4 h, followed by the intestinal environment (pH 6.8) for up to 48 h. The release data showed that BBR was released from the nanocomposite at a higher rate in an acidic environment, with 50.39 ± 3.44% of BBR released after 4 h (Fig. 8). The subsequent introduction of nanocomposites into the intestinal pH media impeded the release process to an extent, with a maximum of 77.96 ± 5.12% of BBR released after 48 h. It should be noted that the maximum release of 73.34 ± 5.54% was achieved after 12 h, and the release became stagnant thereafter with an increment from 73.34 ± 5.54% to 77.96 ± 5.12% during the 12 h to 48 h duration.

## Discussion

BBR-NPs were successfully prepared and characterized for hydrodynamic size and surface charge. The obtained zeta potential value indicates that the surface charge of the nanoparticles is close to zero, which is consistent with the earlier literature reports of nanocarriers possessing PEG-based corona [46, 47]. The tendency to settle down was apparent in the BBR-NPs, possibly because of the absence of surface charge, with both resin components, PEG and PEO, being charge-neutral [35, 48]. The fabrication of neutral surface nanocarriers is imperative in the present case to efficiently penetrate the gastric mucosa and ultimately facilitate gastric absorption of BBR-NPs from the gastric epithelium [37, 49].

To improve the suspensibility of the BBR-NPs in the resin mixture, the presence of PEO in the resin proved to be beneficial, as PEO has been widely reported to improve the stability of dispersion systems [50]. Here, PEO acts as a multifunctional addition to the resin, which provides advantages in both the prototypes’ printability and subsequent controlled BBR delivery.

The nanocomposite pills were printed with excellent printability and reproducibility. The decrease in the mass and volume of the printed pills suggests the removal of water from the nanocomposite drug delivery system during storage. The results from the percent friability and mean hardness suggest the mechanical resilience of the developed nanocomposite drug delivery system. The mechanical properties of the nanocomposite drug delivery system make them suitable for commercial and practical applications.

The excellent swellability can be attributed to the presence of passively entrapped PEO within the photocrosslinked matrix of the nanocomposite, and the excellent hydrophilicity of PEO manifested in the remarkable rehydration or swelling of the composite [51]. The reason for the higher swelling at pH 1.2, compared to pH 6.8, can be attributed to the basicity of the PEO because of the presence of hydroxyl groups which could have facilitated protonation and thus higher solubility as well as swelling at acidic pH [52].

The excellent swelling characteristics of the developed nanocomposites are highly desirable, as higher swelling would result in efficient access across the matrix and its manifestation in the temporal removal of BBR-NPs from the nanocomposite matrix. The nanocomposites exhibited extremely slow degradation with slightly higher mass loss at pH 1.2 as compared to pH 6.8. The swelling was also apparent volumetrically and directed towards the nanocomposite, which is an efficient diffusion-driven drug delivery system.

The lower drug loading in the nanocomposite pill can be attributed to the tendency of BBR-NPs to settle down slowly over the course of the printing process. SEM micrographs further confirmed the entrapment of BBR-NPs within the matrix of the nanocomposite pill. However, the variation between the nanoparticles size obtained from SEM micrograph and DLS The variation between the mean particle size obtained from DLS and electron microscopy can be attributed to the swelling and fusion of BBR-NPs during the SLA process [53].

The faster release at acidic pH shows an agreement with markedly higher swelling under acidic conditions and indicates a swelling and diffusion-driven controlled release of BBR from the nanocomposite drug delivery system [54]. The release data were further fitted with various release kinetics equations to gain insights into the mechanism of drug release from the nanocomposite. Among the different release kinetics models, the release data showed the highest regression coefficient of 0.926 with the Korsmeyer-Peppas model (Table 2) [55]. The results further established the mechanism of diffusion as the driving force for drug release from the nanocomposite, albeit through an intermediate process of diffusion from BBR-NPs. The diffusion coefficient was 0.328 (Table 2), suggesting a pseudo-Fickian diffusion process [56]. The results reflect a complex diffusion process with the drug needing to be released from the BBR-NPs, which in turn should be preceded by their release from the nanocomposite matrix.

**Table 2:**
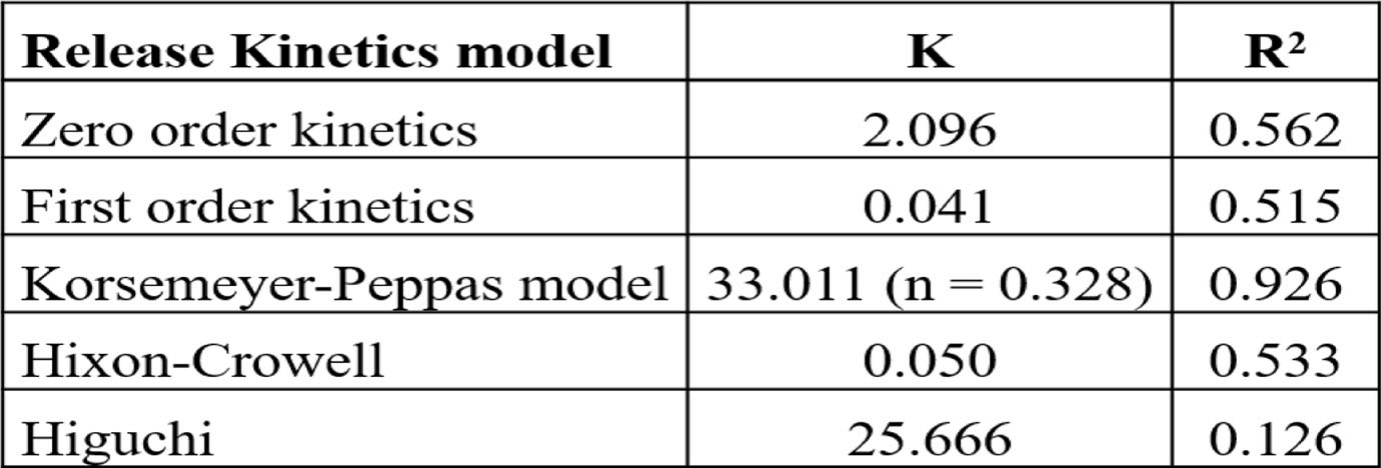
Release kinetic parameters of BBR release when fitted into various release kinetic models

As the drug is released from the nanocomposite based on swelling and diffusion processes, the release pattern can be tailored by modulating the swelling behaviour. Although SLA-based 3D printers are limited by one major parameter, that is, infill density or infill pattern which can be used as a controllable parameter for customized drug release. Hence, to compensate for this limitation, the pills can be printed with varying ratios of the crosslinker (PEGDA to PEG) and by modifying the design of the pill, such as the introduction of a pore-like structure which affects the swelling of the pills.

## Conclusions

The present study aimed to utilise the excellent capabilities of SLA and vat polymerization for achieving 3D printed nanocomposites for medicinal applications. The SLA was utilised for the nanofabrication of BBR-NPs and to embed the prepared nanocarriers into a 3D printed oral dosage form composed of biocompatible and biodegradable components. The loading of BBR onto the NPs and further immobilization of the same in a 3D printed pill provided a sustained release behaviour of BBR. We believe that this approach would be efficacious in improving the gastrointestinal absorption of BBR, reducing its degradation, and improving its bioavailability in vivo. As a proof of concept, the present report provides a perspective that can potentially lead to the development of a novel strategy for preparing SLA-assisted 3DP of composites for a variety of drug delivery applications, including multimodal drug release systems and multi-compartment drug delivery systems.

## Data Availability

The raw dataset can be shared for non-commercial usage based on reasonable requests.

## Abbreviations

SLA: stereolithography
BBR: berberine
NPs: nanoparticles
3DP: three-dimensional printing
CAD: computer-aided design
PEGDA: poly (ethylene glycol) diacrylate
PEO: poly (ethylene oxide)
FTIR: Fourier transform infrared spectroscopy
SEM: scanning electron microscopy
